# Lymphogranuloma Venereum (LGV) Proctocolitis Presenting As Small Bowel Obstruction in an HIV-Negative Man Who Has Sex With Men (MSM) on Pre-exposure Prophylaxis (PrEP): A Diagnostic Dilemma

**DOI:** 10.7759/cureus.111497

**Published:** 2026-06-25

**Authors:** Sneha Adidam, Anna C Pinelo, Pinky Bai, Abay Gobezie, Muhammad Ahmad Imran, Mekdem Bisrat, Naga Sai Shravan Turaga, Farshad Aduli, Huda Gasmelseed

**Affiliations:** 1 Internal Medicine, Howard University Hospital, Washington, D.C., USA; 2 Infectious Disease, Howard University Hospital, Washington, D.C., USA; 3 Medicine, Howard University Hospital, Washington, D.C., USA; 4 Internal medicine, Howard University Hospital, Washington, D.C., USA; 5 Cancer Center, Howard University Hospital, Washington, D.C., USA; 6 Gastroenterology, AdventHealth Orlando, Orlando, USA; 7 Infectious Diseases, Howard University Hospital, Washington, D.C., USA

**Keywords:** chlamydia trachomatis, lymphogranuloma venereum, pre-exposure prophylaxis (prep), proctitis, proctocolitis

## Abstract

Lymphogranuloma venereum (LGV) is an aggressive, sexually transmitted infection caused by specific serovars (L1-L3) of *Chlamydia trachomatis*. Historically considered endemic to tropical regions, LGV has re-emerged in developed nations in recent decades, predominantly affecting men who have sex with men (MSM). Its clinical spectrum is broad, ranging from asymptomatic infection to severe proctitis and proctocolitis, as well as systemic inflammatory manifestations. While buboes were once the hallmark presentation, proctitis syndrome has become the predominant clinical form in contemporary MSM populations, particularly among those engaging in anal receptive intercourse. The increasing use of pre-exposure prophylaxis (PrEP) and associated reduction in condom use have been implicated in facilitating LGV transmission in HIV-negative MSM, expanding the affected demographic beyond those living with HIV. Here, we report a case of a 31-year-old HIV-negative homosexual male patient on PrEP who presented with a two-week history of worsening abdominal pain, fever, nausea, tenesmus, and night sweats. Imaging revealed dilated fluid-filled loops of small bowel consistent with high-grade small bowel obstruction (SBO), prompting an exploratory laparotomy that was unrevealing. Postoperatively, the patient developed a muco-purulent rectal discharge, and further history revealed prior chlamydia infection and a sexual partner with inguinal lymphadenopathy. A rectal swab was positive for *Chlamydia trachomatis* by nucleic acid amplification test (NAAT), and LGV proctocolitis was diagnosed based on high clinical suspicion. Clinical, laboratory, and imaging features are presented in detail. The patient was started on a 21-day course of doxycycline, which led to marked clinical improvement, and his sexual partner was also treated. This case emphasizes the necessity of maintaining a high index of suspicion for LGV in MSM patients on PrEP who present with gastrointestinal symptoms mimicking SBO or inflammatory bowel disease, even in the absence of HIV infection. Prompt recognition and treatment are essential to prevent complications, including rectal strictures, bowel perforation, and unnecessary surgical interventions.

## Introduction

Lymphogranuloma venereum (LGV) is an aggressive, sexually transmitted infection (STI) caused by specific serovars of *Chlamydia trachomatis*. The clinical presentation of LGV ranges from asymptomatic infection to proctitis and proctocolitis [1]. Mild forms of LGV can present as proctitis, while moderate to severe cases can present with fevers, abdominal pain, and tender inguinal lymphadenopathy, also known as buboes [2]. A recent study has shown that proctitis syndrome has become the most common clinical manifestation of LGV, whereas severe presentation in the form of buboes is now uncommon [3].

Proctitis has emerged as an important clinical manifestation in men who have sex with men (MSM), and this is presumably due to the entry of chlamydia pathogens into the rectal mucosa [3]. A 2013 study reported that LGV accounts for 8% of infectious proctitis cases in HIV and 0.7% in HIV-negative men, where it can result in severe hemorrhagic proctitis/proctocolitis [4]. The usual clinical appearance of infectious proctitis can mimic inflammatory bowel disease [5], and a positive nucleic acid amplification test (NAAT) from a rectal swab can help confirm the diagnosis. A 21-day course of doxycycline continues to be the treatment of choice [2] that helps prevent rectal strictures, bowel obstruction, bowel perforation, and other complications [6].

LGV has been recognized as a growing infection in developed countries starting in the early 2000s, and increased frequency was initially reported in Western Europe and most recently in North America [3], where both HIV-positive and HIV-negative MSM populations are affected. The Belgian surveillance data from 2011 to 2017 reported LGV transmission among HIV-negative MSM. This trend may be partly explained by the introduction of pre-exposure prophylaxis (PrEP) and the subsequent decline in condom use [7].

We present a case of an HIV-negative male patient on PrEP, due to multiple male sexual partners, who presented with signs and symptoms of bowel obstruction with no clear etiology other than a confirmed LGV infection. This case report aims to highlight the diverse symptoms and presentation of this infection and the importance of recognizing and treating it promptly to prevent bad outcomes.

## Case presentation

We present the case of a 31-year-old non-HIV homosexual male patient who presented with worsening abdominal pain. He had a two-week history of abdominal pain with nausea and non-bloody emesis. He also reported a fever (101-102°F) with chills, decreased appetite, tenesmus, and night sweats during this time. On examination, the patient’s abdomen was moderately distended and tender in the right upper and lower quadrants, without rebound or guarding. No masses, blood, or any discharge was appreciated on rectal exam. Most of the patient's laboratory results were unremarkable except for significant leukocytosis (14.16). Table 1 compares laboratory values with the references.

**Table 1:** Laboratory results with reference ranges WBC: white blood cell count, HIV: human immunodeficiency virus, RPR: rapid plasma reagin, PCR: polymerase chain reaction

Parameter	Patient value	Reference range
WBC	15.2 x 10⁹/L	4.0-11.0 x 10⁹/L
Hemoglobin	11.6	13.5-17.5 g/dL
Platelets	385	150-400 x 10⁹/L
Sodium	137	135-145 mmol/L
Potassium	4.6	3.5-5.0 mmol/L
Creatinine	0.8	0.6-1.3 mg/dL
Lactate	0.6	0.5-2.2 mmol/L
HIV test	Negative	Negative
RPR	Non-reactive	Non-reactive
Gonorrhea PCR	Not detected	Not detected
Chlamydia PCR (urine/genital)	Not detected	Not detected

CT imaging performed on admission revealed dilated, fluid-filled loops of the small bowel throughout the abdomen and pelvis (Figure 1). A transition zone in the mid-lower left pelvis was seen, attributable to a small-bowel loop with significant wall thickening. Therefore, an NG tube decompression was performed, and IV fluids were started, but the patient showed no significant improvement in symptoms. The patient began to exhibit signs of rebound tenderness and guarding. As a result, the patient was admitted for high-grade small bowel obstruction (SBO) and underwent an exploratory laparotomy. However, the laparotomy was unrevealing. There was no evidence of volvulus, transition point, or inflammation. The patient continued to be conservatively managed for SBO.

**Figure 1:**
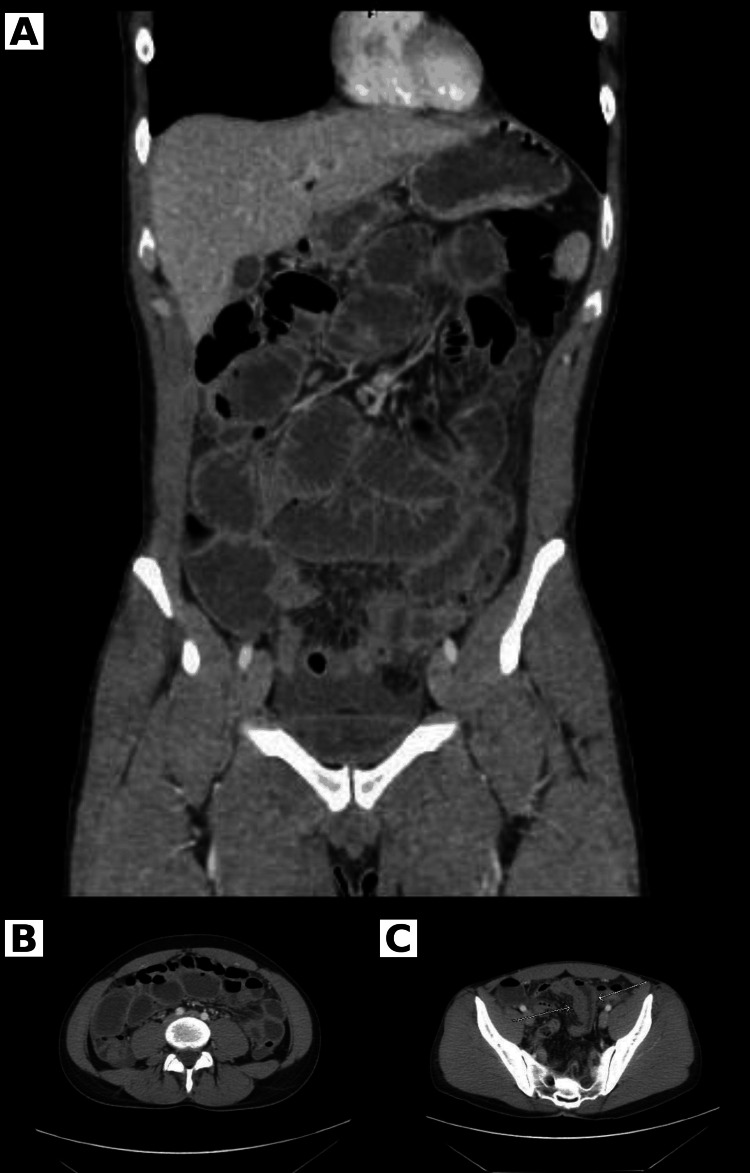
(A, B) CT of the abdomen and pelvis showed dilated fluid-filled loops of small bowel throughout the abdomen and pelvis. (C) A transition zone in the mid-lower left pelvis showing a loop of small bowel with significant wall thickening (arrows) CT: computed tomography

On postoperative day 1, the fever spiked to 103°F. The infectious and STI workup was unremarkable, including negative blood, urine, and stool cultures; negative *Clostridioides difficile*, cytomegalovirus (CMV), and HIV tests; and a negative STI panel (Table 2). However, suspicion for an intra-abdominal infection remained high due to persistent high-grade fever (103°F) and an elevated WBC count (15.2 × 10⁹/L). Therefore, the patient was started on IV ceftriaxone and metronidazole for a five-day course.

**Table 2:** Clinical timeline of symptoms, diagnostic findings, and management CT: computed tomography, SBO: small bowel obstruction, WBC: white blood cell count, CMV: cytomegalovirus, HIV: human immunodeficiency virus, STI: sexually transmitted infection, RPR: rapid plasma reagin, GC: gonococcus (*Neisseria gonorrhoeae*), PCR: polymerase chain reaction, NAAT: nucleic acid amplification test, IV: intravenous

Clinical event/parameter	Week 3	Week 2	Week 1	Admission	Postoperative day 1	Postoperative days 2-5	Discharge
Penile discharge	Yes	-	-	-	-	-	-
Abdominal pain	-	Yes	Yes	Yes	Yes	Improving	Resolved
Fever	-	Yes	Yes	Yes	103°F	Resolving	Resolved
Vomiting	-	Yes	Yes	Yes	-	-	Resolved
CT scan (SBO findings)	-	-	-	Yes	-	-	-
Laparotomy	-	-	-	Yes	-	-	-
Leukocytosis (WBC elevation)	-	-	-	Yes	Yes	Normalizing	Normal
Infectious workup (blood, urine, stool cultures, *Clostridioides difficile*, CMV, HIV)	-	-	-	-	Negative	-	-
STI panel (RPR, GC/chlamydia PCR, trichomonas)	-	-	-	-	Negative	-	-
Rectal discharge	-	-	-	-	Yes	Yes	Resolved
Rectal NAAT (*Chlamydia trachomatis*)	-	-	-	-	Positive	-	-
IV antibiotics (ceftriaxone + metronidazole)	-	-	-	-	Started	Continued	Stopped
Doxycycline therapy	-	-	-	-	Started	Continued	Continued (21-day course)

LGV-specific genotyping (L1-L3) was not performed due to a lack of availability. However, based on high clinical suspicion of proctocolitis, a rectal swab NAAT was obtained and was positive for *Chlamydia trachomatis*.

The next morning, the patient complained of a muco-purulent rectal discharge. Upon further history, the patient revealed his current sexual partner had swollen inguinal lymph nodes, and three weeks before admission, the patient had a penile discharge for seven days, diagnosed as chlamydia, for which he was treated appropriately. During this time, the patient was also on PrEP for unprotected sex with multiple male sexual partners, and his travel history was significant for a trip to Europe and Florida. He had no significant surgical history and denied a family history of inflammatory bowel disease or colorectal cancer.

The patient was empirically started on doxycycline upon the suspicion of *Chlamydia trachomatis* (LGV), and a rectal swab was also sent, which came back positive. The decision was made to treat for proctitis secondary to LGV with a 21-day course of doxycycline. The patient continued to improve and was discharged, and his IV medication was changed to oral (Table 2). His partner was also treated.

## Discussion

The earliest mention of LGV traces back to Wallace (1833), who described what was then considered a "tropical" or climatic bubo in his treatise on venereal diseases. This work marks the first documented clinical description of the condition that would later be recognized as LGV. The clinical and pathological definition of LGV was formally established in 1913 by Durad and Favre [8]. In the index case, there was a strong indication of possible infectious etiology for SBO-LGV proctocolitis. The index patient engaged in anal receptive unprotected intercourse, and the risk factors included sexual preference of MSM, recent travel history, and high-risk sexual practices. In the literature, the incidence is heavily influenced by sexual practices, including anal receptive intercourse, use of foreign objects, recreational drugs, and multiple partners [9].

The incidence and prevalence of LGV proctocolitis are challenging to determine, given that only about half the states in the USA have mandated LGV as a reportable disease to the CDC. There is a consensus that the number of cases, even in industrialized countries, has been increasing since 2003. Before this, Southeast Asia, Africa, and the Caribbean were endemic zones for this disease process [9]. In a study of etiologies of infectious proctitis among HIV-negative men, LGV was found to have a prevalence of 0.7%. Interestingly, another study found that chronic inflammation of colorectal tissue occurs less frequently than acute inflammation in MSM. When chronic inflammatory changes are suspected, evaluation for *Chlamydia trachomatis*, herpes simplex virus type 2, and syphilis should also be considered [10]. Historically, “gay bowel syndrome” was a term used to encompass infectious etiologies in the MSM population. This was considered derogatory and became obsolete [11].

LGV serovars may present in various stages: primary, secondary, or tertiary. The indexed cases had abdominal pain, mucoid discharge per rectum, constitutional symptoms such as myalgia and reactive arthritis, and possible proctitis, stenosis, and proctocolitis [12,13]. This patient presented with high-grade SBO on CT with no surgical evidence of a transition point. CT yields almost 82-100% sensitivity and specificity for diagnosing high-grade obstruction [14]. Spontaneous resolution of SBO occurs in a significant proportion of cases. Only 20% of adhesion-related SBO cases require surgical decompression, with 65-80% successful nonoperative management in acute SBO [15,16]. This patient underwent laparotomy, as there was no improvement with NG tube decompression, and the patient started showing signs of rebound tenderness. There are no published data on spontaneous resolution rates due to non-adhesion or infection in the literature.

The patient was managed with doxycycline 100mg orally twice daily for a total of three weeks and showed marked clinical improvement before discharge. This suggests that infectious colitis/LGV proctocolitis should be treated based on risk factors and a high index of suspicion. Prompt treatment reduces the risk of complications of inflammation, including rectal strictures, bowel obstruction, and perforation [6]. Clinicians should maintain a high index of suspicion for LGV in MSM patients on PrEP with negative baseline STI screening presenting with obstructive gastrointestinal symptoms. Incorporating LGV into the differential diagnosis of SBO-like presentations can help avoid unnecessary surgical interventions and ensure timely initiation of doxycycline.

## Conclusions

This case highlights an atypical and surgically challenging presentation of LGV proctocolitis mimicking high-grade SBO in an HIV-negative homosexual male patient on PrEP. The absence of HIV infection did not preclude severe LGV disease, underscoring that clinicians should not restrict their suspicion to HIV-positive patients when evaluating MSM on PrEP with gastrointestinal symptoms. A positive NAAT rectal swab for *Chlamydia trachomatis*, in the appropriate clinical context, is sufficient to guide empirical treatment even when LGV-specific genotyping is unavailable. Prompt initiation of a 21-day course of doxycycline led to marked clinical improvement and successful discharge, reinforcing that early recognition and treatment are critical to preventing serious complications such as rectal strictures, bowel perforation, and unnecessary surgical intervention. As PrEP use continues to expand and condom use declines among MSM populations, LGV must be incorporated into the differential diagnosis of obstructive gastrointestinal presentations to avoid diagnostic delays and unwarranted laparotomies.
